# Research progress of E-cigarette-A bibliometric analysis during 2010–2022

**DOI:** 10.3389/fpubh.2022.928059

**Published:** 2022-08-01

**Authors:** Shihui Hong, Feng Wu, Wang Yao, Zixuan Yang, Weiguo Wei, Zhe Han, Can Feng, Min Fan

**Affiliations:** Department of Cardiology, Yueyang Hospital of Integrated Traditional Chinese and Western Medicine, Shanghai University of Traditional Chinese Medicine, Shanghai, China

**Keywords:** cigarette, bibliometric, publication, keywords, citation

## Abstract

**Introduction:**

Electronic cigarettes have been widely used all over the world. It is not clear what the advantages and disadvantages of a novelty in daily life are that is attracting increasing attention. Up to now, no bibliometric studies on e-cigarettes have been published in databases. Therefore, we are willing to explore directions and research hotspots in this emerging field by using bibliometrics to analyze research areas, publishing countries and institutions, high-output authors, and future trends of e-cigarettes in recent years. Compared with the traditional review, the bibliometric study can provide some information on core journals, articles, researchers, institutions, and countries concentrating on this topic to guide experimentation strategies and funding decisions.

**Methods:**

A bibliometric analysis was performed by CiteSpace and VOSviewer up to April 2022 in the core collection of Web of Science. HistCite, VOSviewer, CiteSpace, and the R-based Bibliometrix 4.1.0 packages were used to analyze literature information, including year, journal, country, institute, author, keywords, and co-cited references.

**Results:**

Research related to e-cigarettes has proliferated since its inception around 2010. A total of 2,302 studies were published in 689 journals by our search method. Nicotine and tobacco research was the most published journal. The most prolific country was the United States, while the most influential institution was Virginia Commonwealth University. Eight of the top ten authors were from the United States. Oxidative stress, high school students, smoking cessation, delivery, behavioral economics, and exposure were the top topics.

**Conclusions:**

As an emerging social phenomenon, research on e-cigarettes has increased significantly over the past decade, particularly from 2015 to 2020. The top three core journals are Nicotine and Tobacco Research, the International Journal of Environmental Research, and Public Health. Eisenberg-Thomas had published numerous articles on e-cigarettes that had been co-cited in many papers. Oxidative stress, high school students, and smoking cessation are the top three areas of e-cigarette-related research, which were also important areas for further investigation.

## Introduction

An E-cigarette is a rechargeable lithium-polymer battery-powered atomizer that heats the e-liquid (nicotine may or may not be added) in the atomizer oil chamber. E-cigarettes were invented around 2007, and their users have increased exponentially in recent years. Many transnational tobacco companies also began entering the e-cigarette marketplace around 2013. Initially, the original purpose of e-cigarettes was to help people quit smoking, but the convenience of e-cigarettes may have increased the smoking rate of minors and young adults. U.S. high school students' use of e-cigarettes rose 80% in 1 year (2017–2018) (1). The study found that adolescents who had tried e-cigarettes once were far more likely to end up smoking tobacco than those who had never smoked e-cigarette (2). As a result, e-cigarettes have gradually been met with some skepticism from their humble beginnings as a smoking cessation pioneer, with clinicians and public health professionals paying attention and conducting extensive research and discussion on them.

Electronic cigarettes have grown by leaps and bounds recently, especially among young people worldwide. Many electronic cigarette devices have varying qualities, divided into different categories: disposable, rechargeable, and adjustable vaping speed, temperature, and nicotine dose (3). Although some use e-cigarettes as a strategy to quit or reduce smoking, some e-cigarette smokers are still traditional tobacco smokers. They will smoke e-cigarettes as an alternative in places where smoking is inappropriate, potentially increasing their intake of harmful substances. Although e-cigarettes do not contain several toxins released by the combustion of traditional cigarettes, most contain nicotine, a stimulant toxic to the cardiovascular, endocrine, and nervous systems (4). The use of e-cigarettes can lead to smokers being more likely to smoke, especially in China, where indoor smoking is banned, and e-cigarette users are often seen in no-smoking environments such as indoor venues due to their portability and less smoke. The invisibility of use also does make it harder to be subject to public scrutiny.

Given this situation, we have analyzed the research literature related to e-cigarettes in the hope of providing research findings on e-cigarettes. Bibliometrics was introduced by Alan Pritchard in 1969 and is defined as “the application of mathematical and statistical methods to compute and analyze different aspects of textual information to reveal the process of textual processing information and the nature and trends of the development of a discipline” (5). The rapid development of science and technology leads to a sharp increase in scientific literature resources, and it becomes more and more challenging to sort out and analyze the massive literature data. Bibliometrics takes the literature system as the research object, uses statistical analysis, network analysis, and graph theory to study the quantitative relationship, distribution structure, and change law of the literature set, discusses the internal structure of scientific literature, uses quantitative indicators to reflect its quantitative characteristics and regulations, and reveals the internal correlation of resources. Compared with the narrative review, the bibliometric assessment is more objective. Compared with a systematic review, bibliometric methods concentrate on relatively general aspects, such as countries, institutions, authors, and research hotspots, rather than specific viewpoints. A bibliometric study can provide a quantitative overview of a research area. It includes cluster analysis of country and institutional collaboration areas, citation analysis of the literature, co-citation analysis, co-authorship analysis, and keyword analysis. Based on bibliometric techniques, it can explore current research areas and potential future research directions to inform subsequent research (6, 7). Bibliometric analysis can help researchers grasp the primary focus of this field (8). Nowadays, more and more bibliometric analysis articles are public in various journals. But there was still no analysis concerning electronic cigarettes. Therefore, a bibliometric analysis of the e-cigarette was performed to identify the journals and countries in which the literature was published, the institutions that issued the articles, authors, keywords, and the distribution of references of these papers. Our second target was to find out the study trends in this growing field and locate some hot spots to guide the future investigation.

## Methods

### Data sources and search strategy

We conducted a literature search in the Web of Science Core Collection (WoSCC) for the past 12 years (2010–2022) with the keyword e-cigarette. The search strategy: Topic = (electronic cigarette) OR (e-cigarette). The article language was set to English. The WoSCC database is a very well-known database in the medical community. Because it has a large amount of literature citation and citation data, the literature related to bibliometrics in recent years. Most of the literature related to bibliometrics has been done through this database.

### Eligibility criteria and data collection

The literature included in this study for analysis included only articles, excluding conference abstracts and conference proceedings, and the number of papers, citations, titles, authors, institutions, countries, keywords, journals, years of publication, references, and other information were statistically analyzed for bibliometric analysis. Two reviewers (Shihui Hong and Can Feng) independently screened all the literature, annotated, and extracted data from the selected papers, and discussed the literature with disagreement.

### Statistical analysis

A total of four software and software packages were used in this study to perform the package for bibliometric analysis, including HistCite, VOSviewer, CiteSpace, and Bibliometrix 4.1.0 based on the R language.

We have used the Bibliometrix package, a bibliometric analysis tool based on the R language, to analyze leading countries, such as Country Scientific Production and radar map. Visual cluster analysis and timeline visualization of cooperation are formed by VOSviewer, which can present collaboration and temporal trends between countries, institutions, and individuals in graphical images.

The HistCite software provides statistics on the year of publication, country, institution, core journals, h_index and authors, global total citation score (TGCS), and local total citation score (TLCS) for all literature. Cluster analyses are all formed by VOSviewer as well as Scimago graphica.

The Bibliometrix package was used to analyze the source dynamics of core Journals. The dual-map is a typical method of CiteSpace, which can likewise present literature by category, publication time, references, keywords for cluster analysis, biplot overlay analysis, etc.

We have used CiteSpace to cluster co-cited References, present them in chronological order and reveal the most powerful citation bursts. CiteSpace and the Bibliometrix package were used to show the analysis of keywords of co-Cited References.

## Results

### Overall distribution

The WosCC database retrieved 2 302 articles, including 2,055 articles and 247 reviews related to e-cigarettes. Curve fitting analysis showed a general upward trend in the number of annual reports on e-cigarette-related cardiovascular disease since 2010 (*R*^2^ = 0.601). Based on a linear fit, the number of studies will reach ~600 in 2022. This period has been artificially divided into three stages based on annual production and growth rate: the inception stage (2010–2013), the growth stage (2014–2018), and the maturity stage (2019–2022). In the inception stage, the number of articles on e-cigarette-related cardiovascular disease was <20 per year. In 2010, Vansickel AR and Cobb CO, who started working on e-cigarettes, wrote the first article. They did not expose them to measurable amounts but found that they inhibited the assessment of nicotine/tobacco withdrawal symptoms (9). During the same period, Hadwiger attempted to identify aminotarafil and rimonabant in e-cigarette products by diode array high-pressure liquid chromatography and tandem mass spectrometry (cited 68 times) (10).

**Figure 1:**
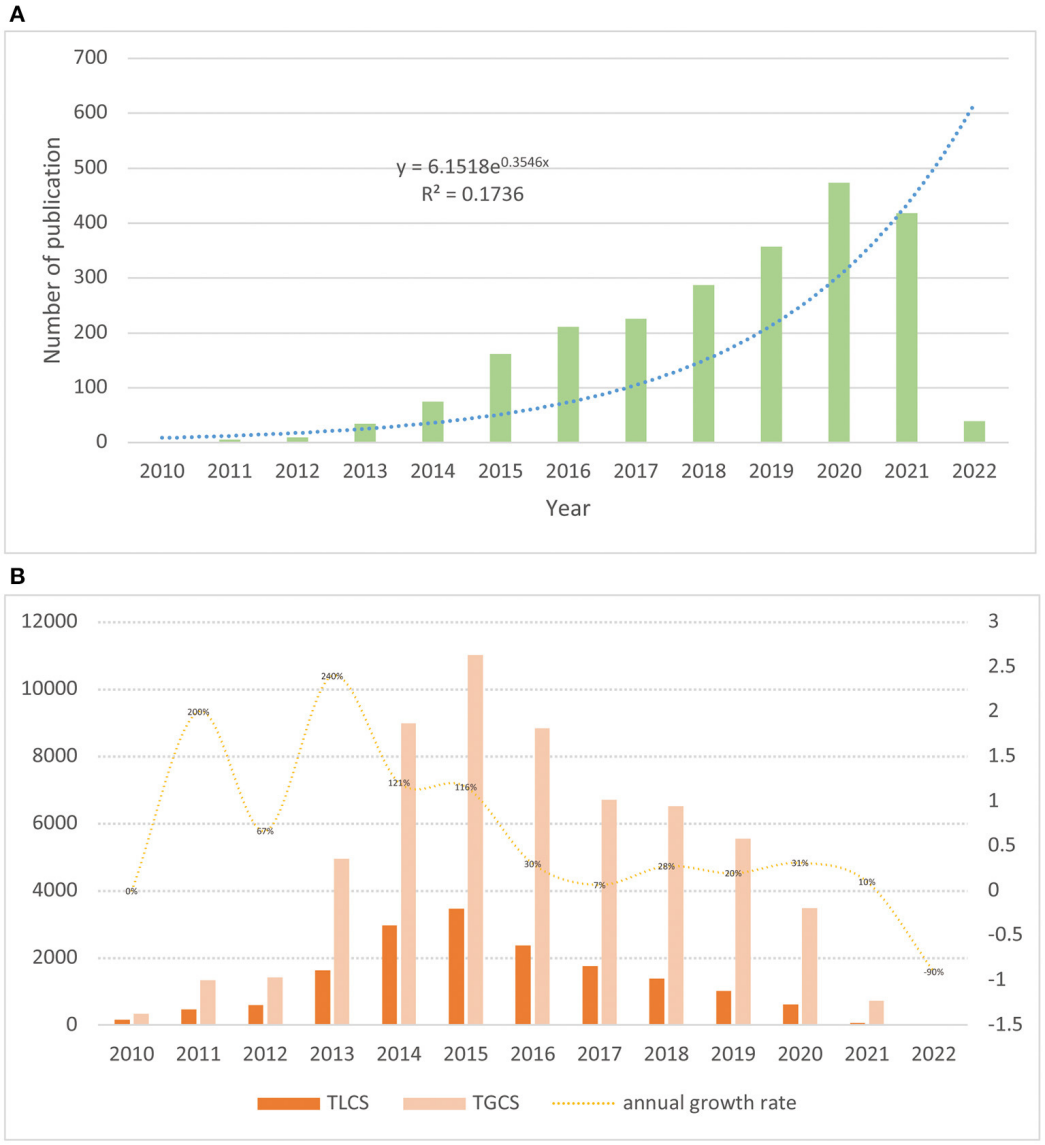
**(A)** Global publication trend analysis of e-cigarette-related publications; **(B)** TGCS, TLCS, and growth rate of e-cigarette literature.

During the growth phase of e-cigarette research, <200 articles were published per year, but the average number of publications increased by about 30 per year, with an average annual growth rate of about 40%. In the maturity phase of the study, the number of articles published per year was >300, with an average annual increase of about 23 articles per year and an average annual growth rate of 17.06% (Figure 1A). The highest number of articles was published in 2020 (*n* = 473).

The selected e-cigarette-related literature in this study had 59,945 citations, with an average of about 26 citations per article. The initial phase literature's TGCS and TLCS were low; from 2013 to 2015, the TGCS increased yearly. By about 2017, the TGCS values were relatively stable, representing that e-cigarette research had entered a relatively mature stage (Figure 1B).

### Leading countries

**Figure 2:**
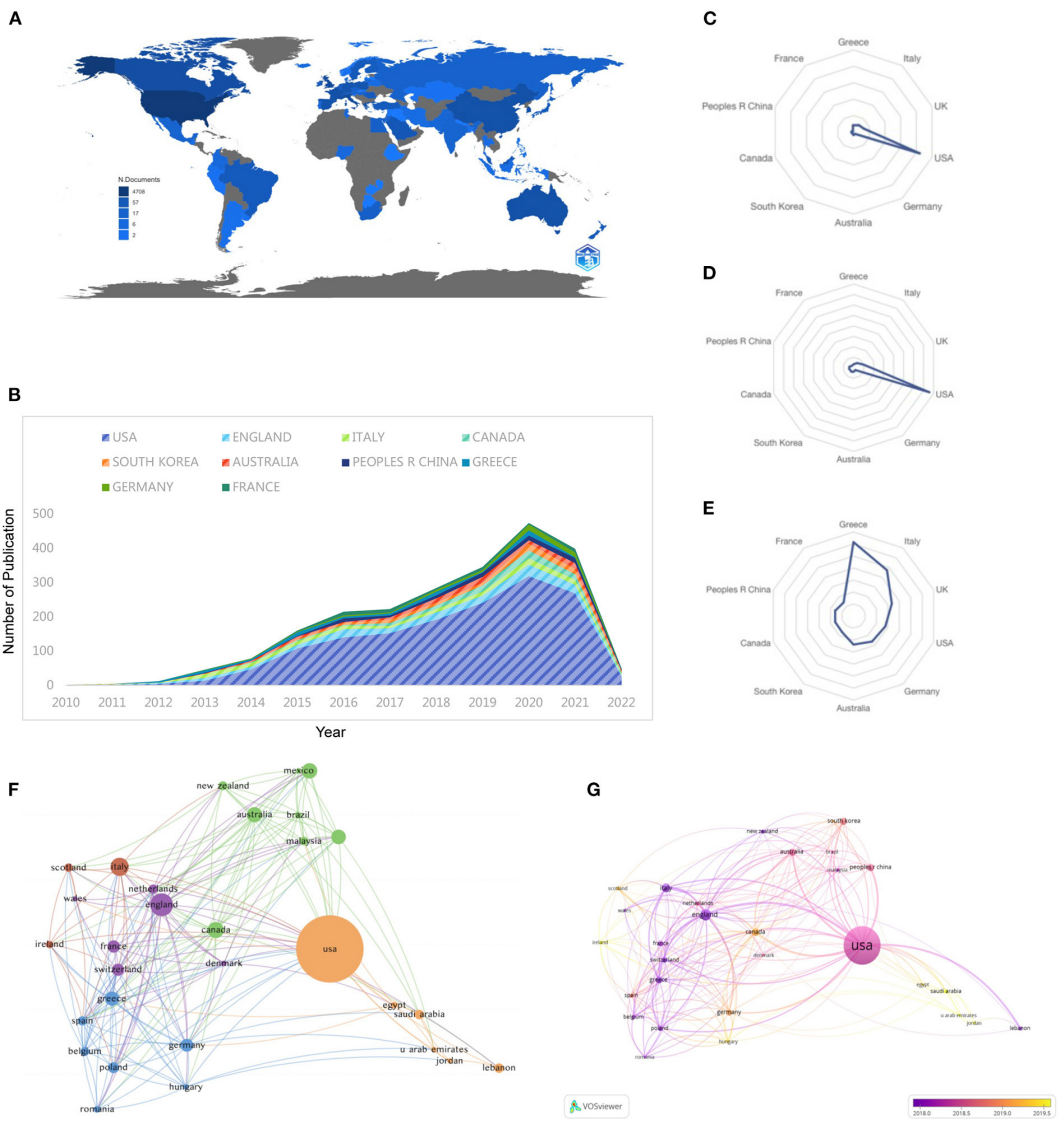
Country analysis of published e-cigarette research literature. **(A)** country analysis of published literature worldwide; **(B)** trends in the number of publications in the top 10 publishing countries over the years; **(C)** radar plot of total literature in the top 10 publishing countries; **(D)** radar plot of TGCS in the top 10 publishing countries; **(E)** radar plot of average citations in the top 10 publishing countries; **(F)** visual cluster analysis of collaboration among publishing countries; **(G)** time of cooperation in each country visualization analysis graphs.

From 2010 to 2022, 79 countries have published research papers on e-cigarettes. Figure 2A shows an overview of the global literature on article generation. The top 10 countries with the highest total number of publications together accounted for ~79% of the worldwide publication volume (Figure 2B and Table 1). Among all countries, e-cigarette literature published in the United States accounted for more than half the number of countries, with a total of 1,515 publications (52.01%); the second-highest number of publications was in the United Kingdom (*n* = 196; 6.73%), and the third was Italy (*n* = 111; 3.81%) (Figure 2C). Corresponding to the number of publications, the most cited country in terms of published articles was also the United States (42,094 citations), followed by the United Kingdom (6,551 citations) and Italy (5,245 citations) (Figure 2D). In addition, Greece had the highest average number of citations in published literature (61.63 citations on average), followed by Italy (47.25 citations on average) and the UK (33.42 citations on average) (Figure 2E), indicating that the literature from these three countries is relatively small and compact. We compared publications between developed and developing countries and did not find a statistically significant difference (*p* = 0.274) (Figure 1A). The visualization of the international cooperation graph shows that the cooperation between countries is close. The United States and China cooperate most closely, while the United States cooperates with all other countries (Figure 2F). Since 2010, research in e-cigarettes began to increase in the United States, the United Kingdom, and Italy, while e-cigarette research in Canada, South Korea, and Australia began to increase mainly after 2015 (Figure 2G).

**Table 1:** Top 10 producing countries for research on electronic cigarette.

**Rank**	**Country**	**Publications *n* (%)**	**LCS**	**GCS**	**Average citation**
1	USA	1,515 (52.01%)	11,320	42,094	27.78
2	UK	196 (6.73%)	1,715	6,551	33.42
3	Italy	111 (3.81%)	1,464	5,245	47.25
4	Canada	87 (2.99%)	280	1,393	16.01
5	Korea	78 (2.68%)	348	1,274	16.33
6	Australia	77 (2.64%)	425	1,830	23.77
7	China	71 (2.44%)	262	1,106	15.58
8	Greece	67 (2.30%)	1,452	4,129	61.63
9	Germany	55 (1.89%)	495	1,443	26.24
10	France	51 (1.75%)	226	713	13.98

GCS, global citation score; LCS, local citation score.

### Active institutes and authors

**Figure 3:**
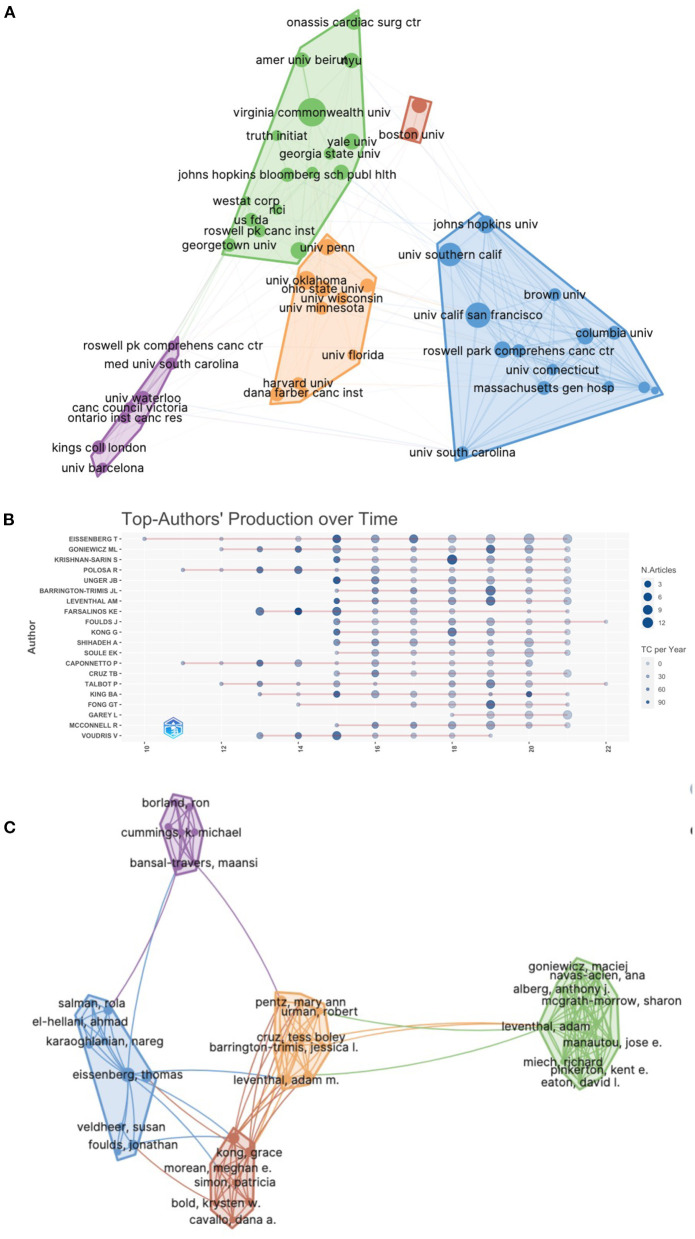
Visual analysis graphs between active institutions and authors. **(A)** cluster analysis between posting institutions; **(B)** timeline analysis of the top 10 posting volume authors; **(C)** cluster analysis of collaboration between authors.

Seven thousand nine hundred ten authors have published articles on e-cigarettes from 2,081 institutions. Table 2 shows the top 10 units with the highest number of publications in e-cigarette studies. The leading units were Virginia Commonwealth University in the USA (*n* = 108), followed by the University of Calif San Francisco in the USA (*n* = 86), the University of Southern Calif in the USA (*n* = 74), and the University of Oklahoma in the USA (*n* = 47). The largest TGCS was that of the University of Calif San Francisco (UCSF) in the USA (cited 5 103 times), followed by Virginia Commonwealth University (mentioned 3,258 times) and the University of Calif San Diego (cited 1,513 times). Cooperation between the organizations was relatively strong. The UCSF-centered group closely collaborates with other institutions (Figure 3A).

**Table 2:** The top 10 productive institutions concerning electronic cigarettes research.

**Rank**	**Institution**	**Publications**	**LCS**	**GCS**	**Average citation**
1	Virginia Commonwealth University	108	1,266	3,258	30.17
2	University of Calif San Francisco	86	1,339	5,103	59.34
3	University of Southern Calif	74	217	1,386	18.73
4	University of Oklahoma	47	243	868	18.47
5	Johns Hopkins University	44	111	640	14.55
6	Ohio State University	38	207	841	22.13
7	University of Calif San Diego	38	437	1,513	39.82
8	University of Michigan	38	165	717	18.87
9	Penn State University	37	344	869	23.49
10	Univ Penn	37	319	1,312	35.46

GCS, global citation score; LCS, local citation score.

The three most productive authors were Eissenberg Thomas from Virginia Commonwealth University (53 papers published), Goniewicz Maciej L from Roswell Park Comprehens Cancer Center (38 articles published), and Krishnan-Sarin Suchitra from Yale University (31 papers published) (Table 3). Eisenberg-Thomas has been involved in a great deal of research on electronic cigarettes and is a senior academic. He began publishing papers on e-cigarettes in 2010 and has continued his research to date (Figure 3B). Goniewicz Maciej L from Roswell Park Comprehensive Cancer Center was the most cited author (cited 2,604 times), followed by Polosa Riccardo from the University of Catania (cited 2,305 times) and Eissenberg Thomas from Virginia Commonwealth University (cited 1,832 times). The level of collaboration between authors was relatively low and characterized by intra-institutional cooperation (Figure 3C).

**Table 3:** The 10 most prolific authors in the field of e-cigarette research.

**Rank**	**Author**	**Country**	**Institution**	**Publications**	**LCS**	**GCS**	**h_index**
1	Eissenberg Thomas	United States	Virginia Commonwealth University	53	793	1,832	19
2	Goniewicz Maciej L	United States	Roswell Park Comprehensive Cancer Center	38	935	2,604	21
3	Krishnan-Sarin Suchitra	United States	Yale University	31	386	1,527	18
4	Polosa Riccardo	ITALY	University of Catania	31	420	2,305	18
5	Unger Jennifer B.	United States	University of Southern California	31	267	1,555	15
6	Barrington-Trimis Jessica	United States	University of Southern California	30	129	1,325	16
7	Leventhal Adam M.	United States	University of Southern California	29	298	1,829	18
8	Farsalinos Konstantinos E.	Greek	King Abdulaziz University	27	970	2,744	22
9	Foulds Jonathan	United States	Pennsylvania State University	26	341	786	13
10	Soule Eric K.	United States	East Carolina University	25	201	464	11

GCS, global citation score; LCS, local citation score.

### Core journals

**Figure 4:**
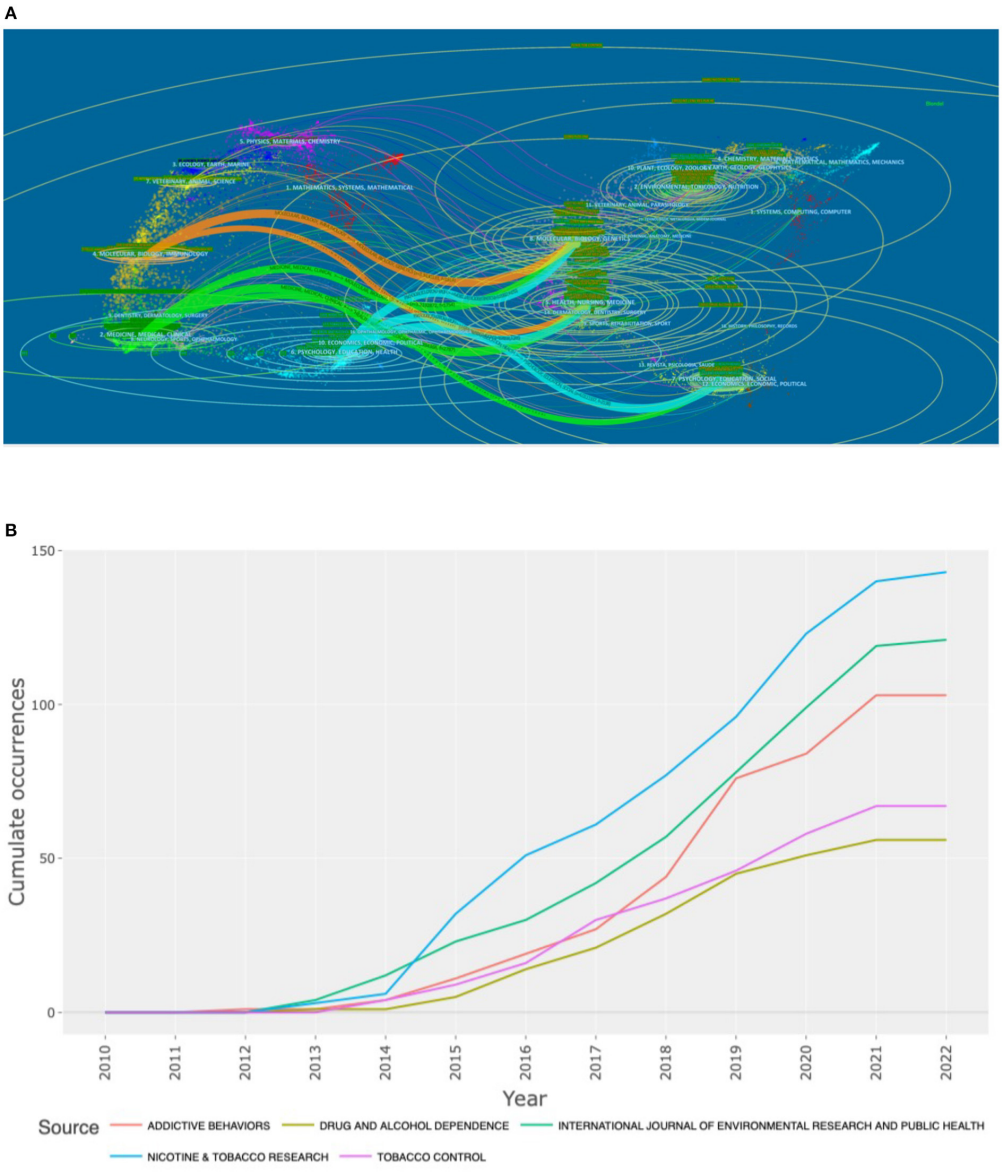
Visualization of Core Journals. **(A)** Souce dynamics of top 5 Core Journals. **(B)** Biplot overlay of article citations for electronic cigarette research.

The articles related to e-cigarette research selected for this study were published in a total of 689 journals. The top 10 most published journals are shown in Table 4, with a total of ~34.94% of the articles published in these journals. The top three most fundamental journals were Nicotine & Tobacco Research (148 articles published, cited 7,044 times), International Journal of Environmental Research and Public Health (121 articles published, cited 3,174 times), and Addictive Behaviors (103 articles published, cited 2,083 times). The annual number of articles published in each journal has been increasing yearly. The average annual growth rate of Nicotine & Tobacco Research publications was 78.59% over 2010–2022 (Figure 4A).

**Table 4:** The top 10 core journals on electronic cigarettes research.

**Rank**	**Journal**	**Recs**	**LCS**	**GCS**	**IF (2022)**	**H-index**
1	Nicotine & Tobacco Research	148	2,637	7,044	3.427	40
2	International Journal of Environmental Research and Public Health	121	982	3,174	2.389	27
3	Addictive Behaviors	103	699	2,083	3.558	24
4	Tobacco Control	77	1,022	3,094	5.231	31
5	Drug and Alcohol Dependence	56	545	1,649	4.066	23
6	PLoS One	52	0	2,529	3.041	26
7	Addiction	43	1,021	2,643	3.689	23
8	Pediatrics	41	253	2,237	4.106	21
9	Journal of Adolescent Health	36	446	1,545	3.260	20
10	Preventive Medicine	35	218	831	3.102	20

IF, impact factor; TGCS, total global citation score; TLCS, total local citation score; H-index, Hirsch index.

The citation and cited status of journals reflects the thematic distribution (Figure 4B); cited journals are on the left, cited journals are on the right, and the colored paths represent citation ratios (11).

### Co-cited references

The more cited studies have focused on smoking cessation and smoking among adolescents. The most widely cited article to date is the one by Goniewicz ML on the significant reduction in population exposure to specific tobacco toxicants by replacing tobacco with e-cigarettes (cited 980 times) (Table 5) (12). Grana R then conducted a scientific review of e-cigarettes and proved that e-cigarette emissions are not just “harmless water vapor” but a source of indoor air pollution (cited 803 times) (3). We then created a visualization network of cited references and performed a cluster analysis. In total, we found 12 clusters, with an average silhouette value of 0.877 (Supplementary Table 1). The silhouette value measures how similar an object is to its collections compared to other clusters. The silhouette value ranges between [1, −1], with higher values representing good similarity to its clusters and poor similarity to neighboring clusters.

**Table 5:** Top 10 most cited papers.

**Rank**	**Author**	**Source**	**Year**	**IF (2022)**	**Category**	**Cluster ID**	**TLCS**	**TGCS**	**Centrality**
1	Goniewicz ML	TOB Control	2014	5.231	Tetrahydrocannabinol	3	381	980	0.03
2	Grana R	Circulation	2014	9.484	Smoking cessation	0	255	803	0
3	Leventhal AM	Jama-J Am Med Assoc	2015	11.381	Adolescent smoking	2	237	795	0.02
4	Bullen C	Lancet	2013	14.806	Smoking cessation	0	223	504	0.03
5	Soneji S	Jama Pediatrics	2017	3.786	Adolescent smoking	2	219	594	0.01
6	Caponnetto P	PLoS ONE	2013	3.041	Smoking cessation	0	209	460	0.06
7	Adkison SE	Am J Prev Med	2013	3.997	Smoking cessation	0	204	479	0.01
8	King BA	Nicotine TOB Res	2015	3.427	Smoking cessation	0	201	510	0.01
9	Kosmider L	Nicotine TOB Res	2014	3.427	Tetrahydrocannabinol	3	198	575	0.03
10	Kalkhoran S	Lancet Resp Med	2016	4.663	Smoking cessation	0	175	394	0.04

**Figure 5:**
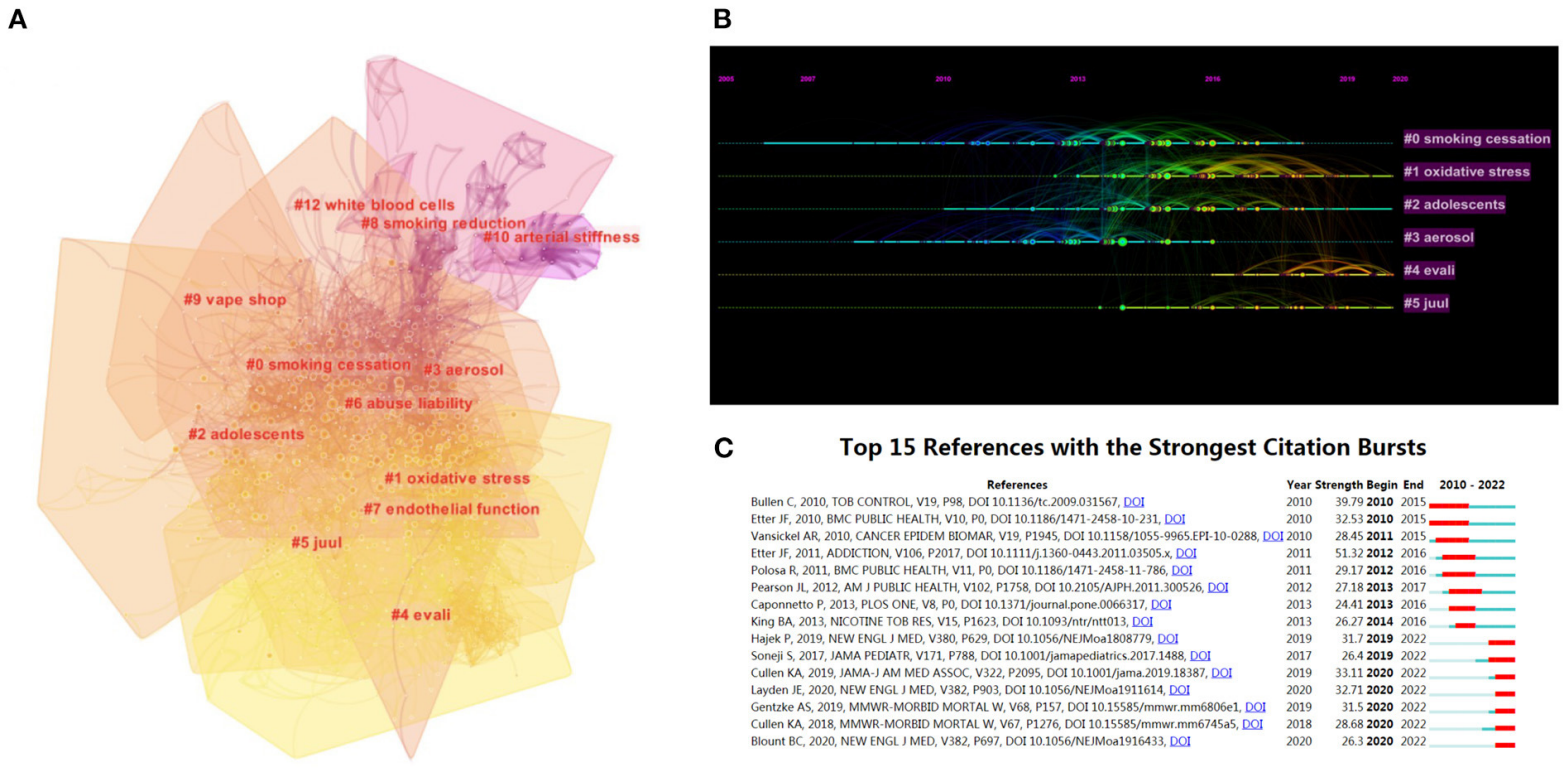
Visual analysis of co-cited reference analysis. **(A)** Cluster analysis graph of co-cited references; **(B)** top 6 clusters and time and intensity distribution; **(C)** table of the start and end time and power of citation outbreaks for the top 15 references in citation outbreak intensity.

Co-cited references are those references that have been co-cited in other publications (13). In this scientometric review, the six largest clusters of associated references were selected to define the knowledge base on the electronic cigarette. The six clusters with the highest K values were identified (Figure 5A and Table 6), which include “smoking cessation,” “oxidative stress,” and “adolescents,” among others. In addition, we performed a visualized timeline for the clusters (Figure 5B). We found that “smoking cessation” is an early domain of e-cigarettes. However, the current hotspots of electronic cigarettes are “oxidative stress,” “heavy metals,” and “tetrahydrocannabinol.”

**Table 6:** The 6 highest K-value co-cited reference groups.

**ClusterID**	**Size**	**Silhouette**	**Mean (Year)**	**Top term**
#0	180	0.722	2013	Smoking cessation
#1	140	0.808	2016	Oxidative stress
#2	123	0.862	2015	Adolescents
#3	110	0.792	2012	Aerosol
#4	107	0.899	2018	Tetrahydrocannabinol
#5	65	0.877	2017	Heavy metals

Finally, we carried out a reference explosion. Citation bursts are those references that are frequently studied in detail by scientists in each field over a given time interval (14). Figure 5C shows the time of publication, intensity, and time of origin of the 15 references with the most robust citation bursts. As seen in the figure, Bullen's work has the highest burst intensity (39.79) (15). He measured the effects of e-cigarettes on smoking addiction, withdrawal symptoms, acceptance, pharmacokinetic properties, and side effects. In addition, Benjamin C. Blount's paper has been heavily cited in recent years (16). In his paper, he explores the causative factors of E-cigarette or Vaping Use-Associated Lung Injury (EVALI) that are currently occurring in some areas with severe hazards and finds that this side effect of e-cigarettes has attracted increasing research in recent years. The top 25 most frequently cited references are shown in Supplementary Figure 2.

## Analysis of keywords

**Figure 6:**
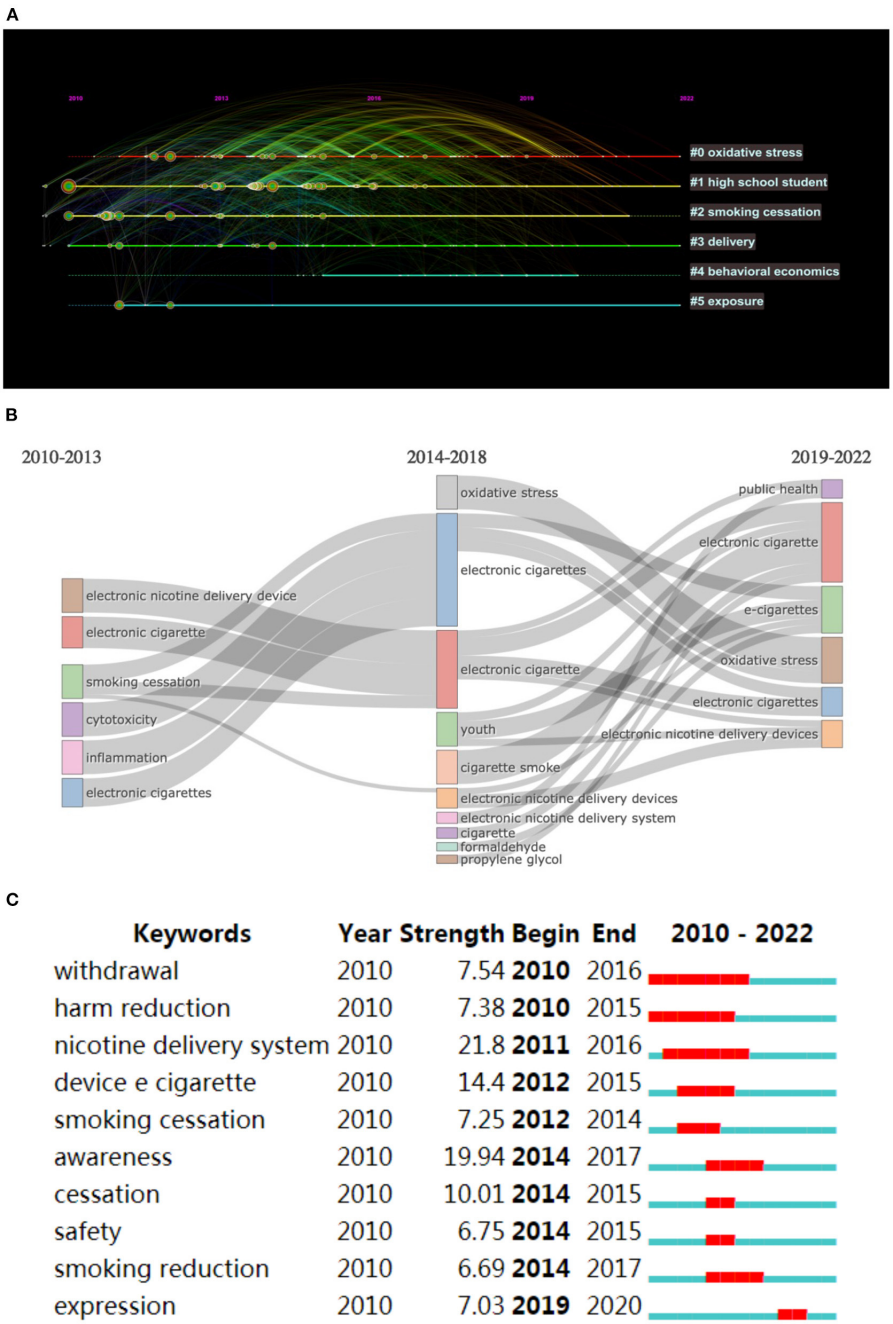
Visual analysis of keywords. **(A)** timeline analysis after keyword clustering; **(B)** keyword Sankey evolution diagram of e-cigarette research; **(C)** representative burst keywords in the top 10 references.

**Figure 7:**
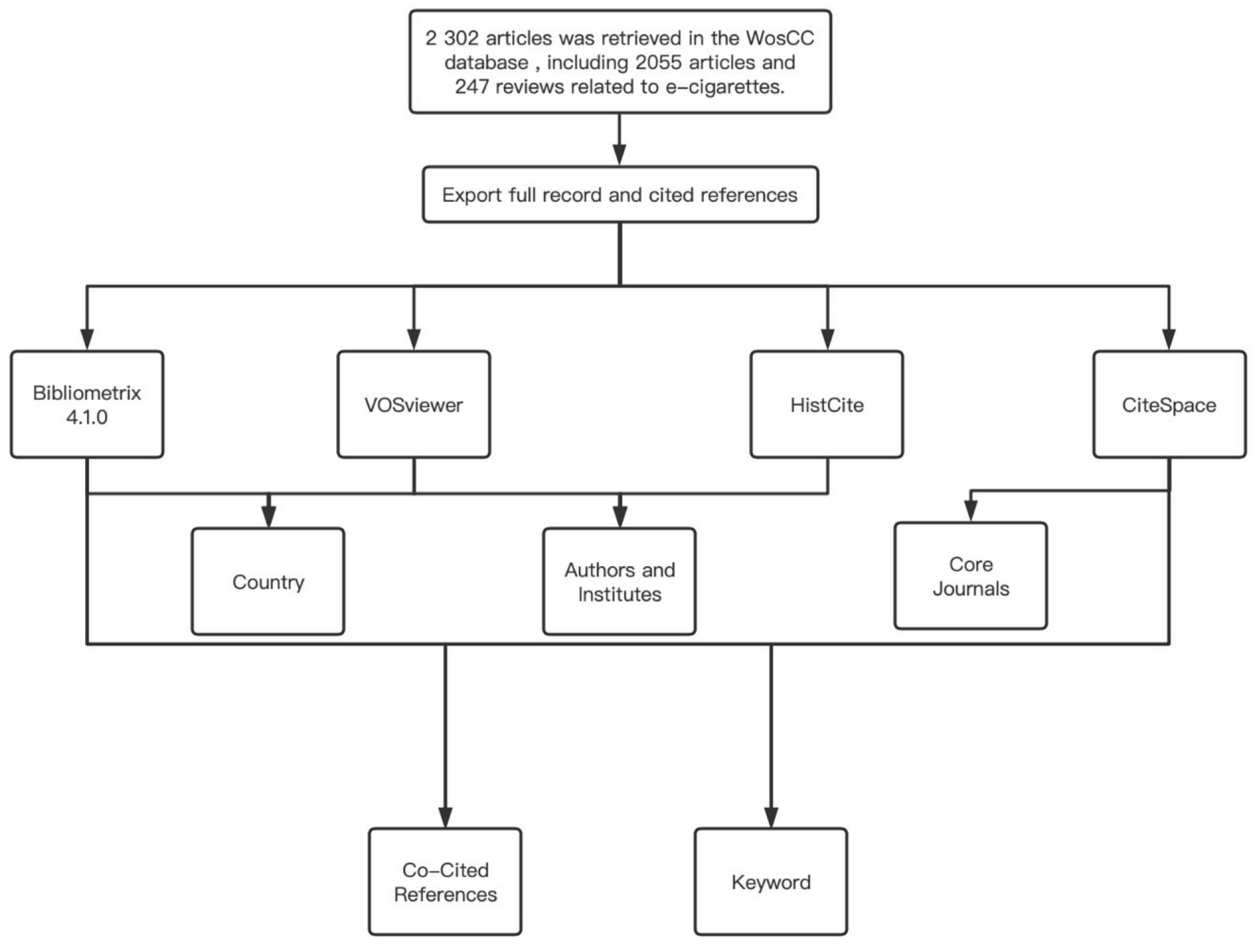
Methods flow chart.

We collected and analyzed a total of 3,144 keywords from the literature and obtained 8 clusters by cluster analysis (Q modulus of 0.4049 and mean silhouette value of 0.643, Table 7). The clustering results showed that “oxidative stress” and “high school students” were the two most essential areas in e-cigarette research, while “delivery” was a persistent hot spot (Figure 6A). We also performed a keyword evolution analysis and found that the initial phase of e-cigarette research focused on “electronic nicotine delivery devices.” However, as the research field has matured, the main research focus on electronic cigarettes has gradually changed to “oxidative stress.” In the last 3 years, “youth,” “public health,” among others, have gradually attracted the attention of scientists (Figure 6B). In the past 3 years, subordinate directions such as “youth” and “public health” have gradually attracted the attention of the academic community (Figure 6B). The 25 keywords with the highest intensity were obtained by keyword blast citation analysis (Supplementary Figure 3). We found that the term “nicotine delivery system” had the highest burst intensity. In addition, we also found that “expression” was the most frequent keyword in the last 2 years (Figure 6C). All our analyses can be summarized in the methods flow charts (Figure 7).

**Table 7:** Keyword clustering analysis of e-cigarette research.

**Cluster ID**	**Size**	**Silhouette**	**Mean (Year)**	**Top terms**
#0	179	0.581	2017	Oxidative stress
#1	149	0.564	2016	High school student
#2	103	0.594	2014	Smoking cessation
#3	66	0.7	2015	Delivery
#4	24	0.805	2017	Behavioral economics
#5	19	0.956	2012	Heart disease
#6	17	0.936	2012	Smoker
#7	15	0.927	2020	Vitamin e acetate
#8	14	0.946	2017	Oral commensal bacteria
#9	6	0.96	2018	Tobacco policy
#10	4	0.995	2012	Tobacco-related disease

## Discussion

The significance of bibliometric analysis is to understand the history of the field of research, grasp the present, and guide the future. In this study, we analyzed the characteristics of previously published literature on e-cigarettes, including countries, institutions, authors, and keywords. We looked at possible future research trends through a bibliometric analysis. We searched the WoSCC and found a total of 2,302 e-cigarette-related publications, mainly published from 2010 to 2022, with an average of about 192 publications per year. Between 2010 and 2014, the number of articles published each year was <100. After 2015, the number of relevant articles began to increase significantly. By about 2020, the number of published articles will be close to 500 per year. This trend is related to the increasing interest in e-cigarettes, which are becoming popular worldwide. This trend reflected concerns from governments, medical institutions, epidemiologists, and respiratory physicians.

The 2,302 articles were published by 2,081 institutions in 79 countries, with 1,822 (79.14%) of the papers published by the United States, the United Kingdom, and Italy, suggesting that these three countries have played an essential role in driving the development of e-cigarette research. The United States is the leader in e-cigarette research, with far more publications than any other country occupying the most significant number of articles, with Virginia Commonwealth University publishing the most of any institution, and their team in the Department of Psychology conducting a great deal of in-depth research and scientific collaboration in the field of e-cigarettes. China and the United States had the most partnerships among the countries that collaborated. This highlights the contemporary trend of scientific cooperation between large countries. These findings indicated that they paid more attention to e-cigarettes. Even though e-cigarettes were produced for smoking cessation, they also had side-effects.

Eisenberg-Thomas is a leading scholar in e-cigarettes in the United States and has published extensively on e-cigarettes, covering the study of e-cigarette regulatory policy (17–19). Most of his articles warned people of the adverse effects of e-cigarettes and suggested policy regulation accordingly (20–22).

About 1/3 of the literature on e-cigarettes had been published in the top 10 journals. Primarily the top 3 journals had published more than 100 papers. The top three journals were Nicotine & Tobacco Research, International Journal of Environmental Research and Public Health, and Addictive Behaviors. All three journals focus on the behavior and impact of electronic cigarettes. For example, a recent study published in Nicotine and Tobacco Research found that different nicotine concentrations and more decadent flavors may lead to higher e-cigarette use (3).

In science, it is essential to stand on the shoulders of giants. Thus, highly cited literature implies common recognition and learning. The most cited article was about the benefits of electronic cigarettes, which had 980 times citation (12). This is also the original purpose of the production of electronic cigarettes. Due to the profit-seeking nature of manufacturers, expanding production and sales is the inherent goal of enterprises, so the subsequent development gradually exceeded the original intention of the actual electronic cigarette. Then a critical review was published about the “harmless water vapor” source of indoor air pollution (3). This review enlightened the studies in this field.

Cluster analysis also gave us much information about the main topic in the spotlight. First, smoking cessation is the most concerning point in the eye of researchers, which is also the e-cigarette's primary function. Then the oxidative stress caused by e-cigarettes has the same effect as traditional cigarettes. The third cluster was adolescents. Many people may notice that due to the small size, portability, and ease of use, the e-cigarette is gradually becoming welcomed among adolescents. The remaining three clusters focus on the volatile components and hazards of e-cigarettes, and this is the area where we can focus our research efforts.

Among the top 10 most cited articles, 60% (6/10) had smoking cessation as the primary research content, indicating that smoking cessation is still the principal value of e-cigarettes and a research hotspot in academia. Besides, adolescent smoking was also a significant concern in this field. Goniewicz found that among smokers who were unwilling to quit altogether, using e-cigarettes instead of traditional tobacco could reduce the harm caused by smoking (12). Grana published a comprehensive review in cardiology's top journal, CIRCULATION, that comprehensively summarizes nearly everything about e-cigarettes (3). Publications with intense citation bursts represent that the article has been widely cited by researchers over a time period (23). Time-line tracking reveals the research trends of scholars on e-cigarettes in different periods. It began with device-based studies when e-cigarettes were first introduced, then progressed to reflections on the hazards of oxidative stress damage from e-cigarettes, it has focused on the effects on adolescent populations and public health. The citation bursts of the top eight publications end around 2015. Then seven articles opened the 2019–2022 citation explosion, among which two were published in JAMA and another two in The New England Journal of Medicine (16, 24).

As research evolves, several emerging research areas become subjects of interest to researchers. Burst detection is a method used to identify sudden increases in the frequency of keywords and references appearing in a certain period and can identify research hotspots and important research literature. References and keywords outbreaks show that some projects have seen the highest outbreak intensity in the last 3 years. At first, the electronic cigarette had been implied to be a suitable device for smoking cessation alternative (15), and also some surveys about electronic cigarettes (25). A large amount of data on the benefits of e-cigarettes continues to be reported, increasing the acceptance of e-cigarettes (9, 26). With behavioral support, smoking cessation rates were higher with e-cigarettes than with traditional nicotine replacement therapy (27). Once adolescents and young adults have a history of e-cigarette use, subsequent rates of smoking traditional cigarettes are higher (28).

Keyword clustering analysis and burst detection also reflect recent research hotspots (29). Keywords are also the concentration of hot spots. Three thousand one hundred forty-four keywords were analyzed and divided into 8 clusters. The top 3 clusters contained more than 100 keywords. The results showed that “oxidative stress” and “high school student” received the most attention. The first one reflects the negative pathophysiology consequences of electronic cigarette smoking. An E-cigarette is new, so most researchers concentrate on its effect on people's health. The high school student population is the most vulnerable to the attraction and addiction to e-cigarettes; they are underage and easily influenced by advertisements, TV, and movies to start smoking e-cigarettes or regular cigarettes. To our knowledge, e-cigarettes are not an entirely healthy substitute for traditional smoking, especially for cardiovascular disease (30).

Daily electronic cigarette use is independently associated with increased HR and BP (31) and myocardial infarction risk (32).

The effects of electronic cigarettes on hemodynamics could be the result of the heavy metals or other unknown ones which contain adverse effects on vascular function and hemodynamics.

Notably, e-cigarette-associated pneumonia is likely to be a significant research trend in the future. It is necessary to find relevant cases, etiology, and solutions to avoid causing more harm to electronic cigarette users.

## Strengths and limitations

To our knowledge, this is the first study to analyze e-cigarettes research using a bibliometric approach. In contrast to traditional reviews, bibliometric analysis allows for the analysis of evolving research priorities and trends from a point in time. It enables the identification of essential articles as well as researchers and institutions.

Our study also has some limitations. First, literature published outside the WoSCC database is missed, resulting in research bias. Second, most of the results in this study were based on machine algorithms and were slightly deficient in manual generalization. Third, all the included e-cigarette studies were published in English. It is possible that e-cigarette-related literature published in other languages was overlooked.

## Conclusion

The number of papers on e-cigarettes has increased year by year. We used bibliometric methods to analyze research on e-cigarettes over the past decade, with the United States contributing the most; Virginia Commonwealth University had the most publications among institutions. The top three core journals were Nicotine and Tobacco Research, International Journal of Environmental Studies, and Public Health. Eisenberg-Thomas published the most articles. Oxidative stress, high school students, smoking cessation, and tetrahydrocannabinol are the knowledge base for e-cigarette-related research. As research progresses, we expect that e-cigarettes will serve the purpose of smoking cessation and radically reduce the health damage caused by cigarettes.

## Data availability statement

The original contributions presented in the study are included in the article/Supplementary Material, further inquiries can be directed to the corresponding authors.

## Author contributions

WY and MF contributed to conception and design of the study. ZY organized the database. SH performed the statistical analysis. CF wrote the first draft of the manuscript. ZH, WW, and FW wrote sections of the manuscript. All authors contributed to manuscript revision, read, and approved the submitted version.

## Funding

This work was supported by the National Natural Science Foundation of China (NSFC) grant (82074266, 81904007), Science and Technology Commission of Shanghai Municipality (STCSM) Research Fund (21JC1405300, 202040306) and Yueyang Hospital Research Projects (2018YJ05).

## Conflict of interest

The authors declare that the research was conducted in the absence of any commercial or financial relationships that could be construed as a potential conflict of interest.

## Publisher's note

All claims expressed in this article are solely those of the authors and do not necessarily represent those of their affiliated organizations, or those of the publisher, the editors and the reviewers. Any product that may be evaluated in this article, or claim that may be made by its manufacturer, is not guaranteed or endorsed by the publisher.
