# Supplementary material for: Research progress of E-cigarette-A bibliometric analysis during 2010–2022
Source: Front Public Health. 2022 Aug 1;10:928059. doi: 10.3389/fpubh.2022.928059 (PMC9376676; doi:10.3389/fpubh.2022.928059)
Supplement: Supplementary file 1 [file Data_Sheet_1.docx]

Supplementary Material

# Supplementary Figures and Tables

## Supplementary Figures


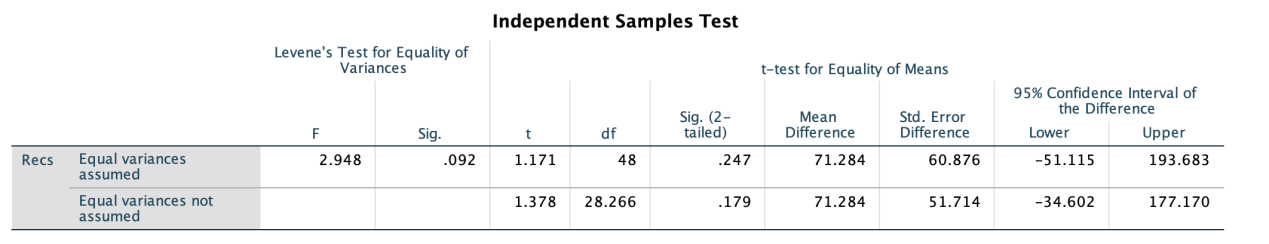


**Supplementary Figure 1.** Comparison of publications between developed and developing countries


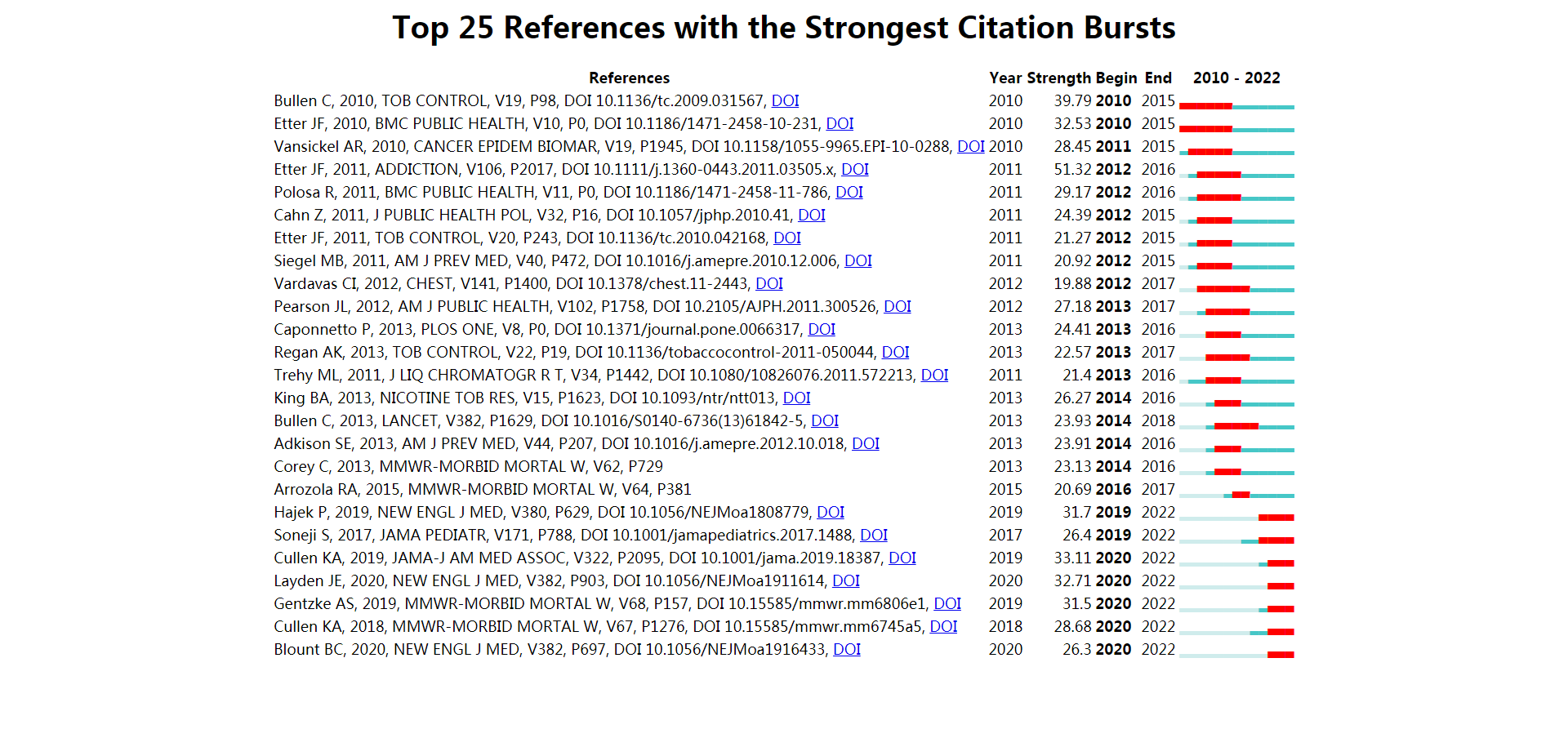


**Supplementary Figure 2.** Top 25 references with the strongest citation bursts


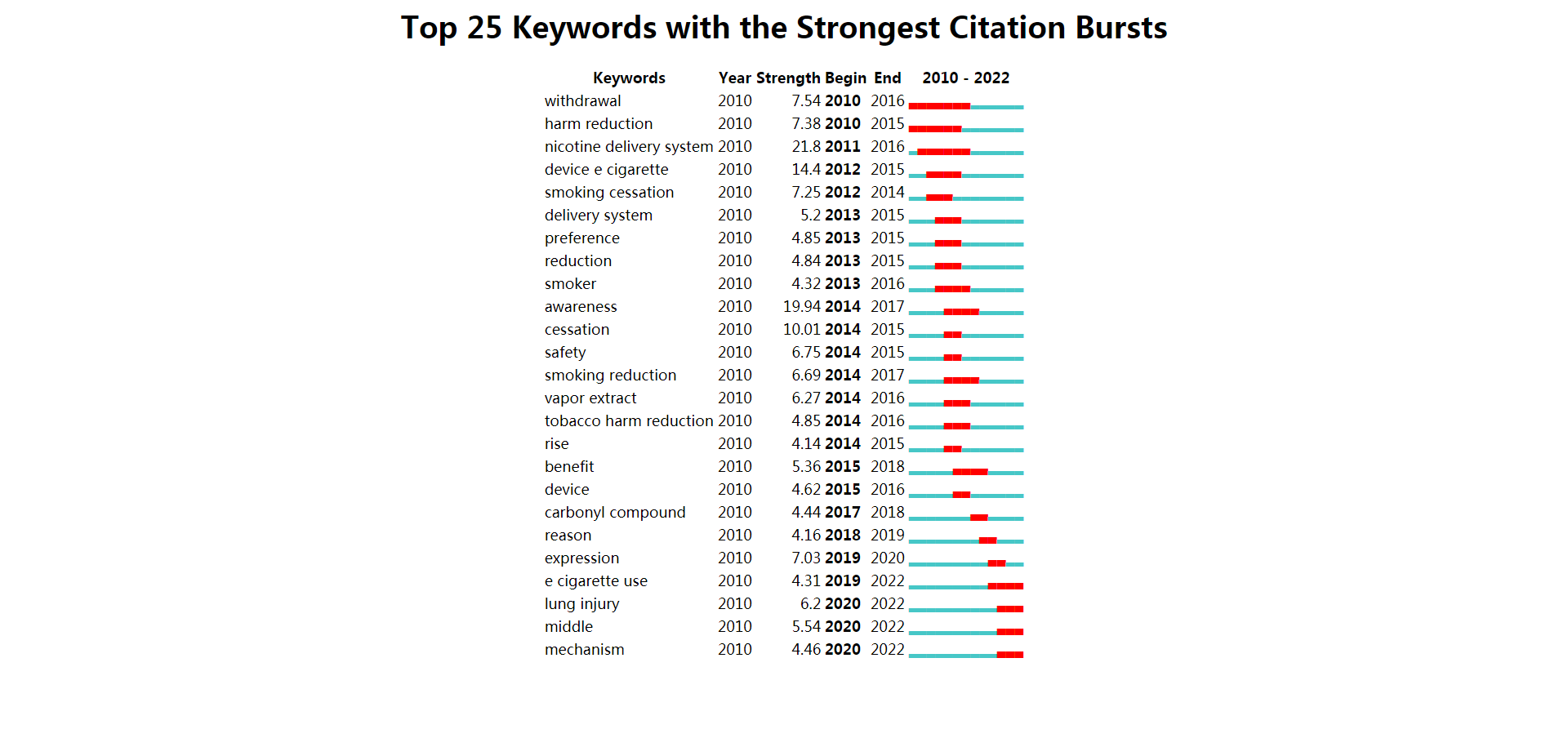


**Supplementary Figure 3.** Top 25 keywords with the strongest citation bursts

**Supplementary Table 1.** Co-cited references cluster analysis of electronic cigarettes research.

| ClusterID | Size | Silhouette | mean(Year) | Top term |
| --- | --- | --- | --- | --- |
| #0 | 180 | 0.722 | 2013 | smoking cessation |
| #1 | 140 | 0.808 | 2016 | oxidative stress |
| #2 | 123 | 0.862 | 2015 | adolescents |
| #3 | 110 | 0.792 | 2012 | aerosol |
| #4 | 107 | 0.899 | 2018 | tetrahydrocannabinol |
| #5 | 65 | 0.877 | 2017 | marketing |
| #6 | 58 | 0.817 | 2014 | abuse liability |
| #7 | 57 | 0.893 | 2017 | cardiovascular disease |
| #8 | 50 | 0.937 | 2009 | harm reduction |
| #9 | 34 | 0.939 | 2014 | young adult |
| #10 | 24 | 0.987 | 2008 | e-cigarette associated lung injury |
| #12 | 8 | 0.991 | 2009 | white blood cells |
